# A systematic review of educational interventions to enhance ethical sensitivity in nursing students in Asia and the Middle East

**DOI:** 10.1186/s12910-025-01334-x

**Published:** 2025-12-30

**Authors:** Xiaopu Shi, Rui Wang, Ying Li, Lili Zeng

**Affiliations:** 1 School of Life Science and Health, Huzhou College, Huzhou, Zhejiang 313000 China; 2 Department of Health Management, Heze Medical College, Heze, China; 3 Huzhou Zhebei Mingzhou Hospital, Huzhou, China

**Keywords:** Nursing students, Ethical sensitivity, Educational intervention, Systematic review

## Abstract

**Background:**

As future providers of medical services, nursing students require strong ethical sensitivity to effectively manage clinical ethical dilemmas. However, studies have shown that they often exhibit lower ethical sensitivity than practicing nurses, which may hinder their ability to address ethical challenges competently.

**Objective:**

This systematic review aimed to comprehensively evaluate the effectiveness of educational interventions in enhancing the ethical sensitivity of nursing students. The findings will offer practical guidelines and a theoretical foundation for advancing nursing ethics education.

**Methods:**

This review adhered to the Preferred Reporting Items for Systematic Reviews and Meta-Analyses (PRISMA) guidelines. We conducted comprehensive searches across nine databases (PubMed, Embase, Medical Literature Analysis and Retrieval System Online (MEDLINE), Web of Science, Cochrane Library, Cumulated Index in Nursing and Allied Health Literature (CINAHL), China National Knowledge Infrastructure (CNKI), VIP (Chinese Science and Technology Journal Database), and Wanfang Data) to identify relevant studies published through December 2024. Two researchers independently performed the screening, data extraction, and quality assessment, with any disagreements resolved through discussion to reach a consensus.

**Results:**

Twenty-one studies were included. The results indicated that current educational interventions primarily focus on two dimensions: teaching methods and curricular content. Among teaching methods, situational participatory learning was found to be more effective than traditional didactic instruction in enhancing nursing students’ ethical sensitivity. The curricular content was organized into a three-dimensional framework encompassing fundamental theories, clinical practice, and interdisciplinary integration, establishing a comprehensive knowledge system ranging from ethical principles to emerging technological ethics.

**Conclusions:**

This review confirmed that interactive teaching methods effectively enhance nursing students’ ethical sensitivity, although they require significant resources. Future nursing education should integrate cross-cultural competencies, spiritual care training, and emerging ethical issues while establishing comprehensive ethics curricula within clinical training. Such approaches will better prepare nurses for ethical challenges in practice.

**Supplementary Information:**

The online version contains supplementary material available at 10.1186/s12910-025-01334-x.

## Introduction

 Advances in medical technology have led to increasingly complex ethical dilemmas in clinical nursing practice. As integral members of healthcare teams, nurses routinely encounter morally challenging situations. Research indicates a high prevalence of ethical issues, with 46.7% of intensive care unit nurses reporting exposure to such dilemmas and 35.7% reporting difficulties in resolving them [1]. This contributes significantly to professional uncertainty and moral distress. Furthermore, emerging technologies such as artificial intelligence (AI), big data, and robotics have introduced new ethical challenges. There are concerns including algorithmic bias in clinical decision support tools and privacy risks associated with digital health platforms [2, 3], necessitating heightened ethical sensitivity to both traditional and technology-driven dilemmas.

Ethical sensitivity, defined as an individual’s ability to perceive ethical dilemmas and commit to ethical principles [4], is essential to core nursing competencies. Nurses with higher levels of ethical sensitivity are better equipped to identify and address ethical issues, thereby enhancing care quality [5]. As future practitioners, nursing students will inevitably encounter high-risk ethical scenarios that require sound decision-making. However, studies have shown that their ethical sensitivity levels are generally lower than those of practicing nurses [6], raising concerns about their preparedness for clinical ethics.

Consequently, ethics education is recognized as fundamental to the development of ethical sensitivity and constitutes a core component of nursing education [7]. The increasing complexity of ethical conflicts in clinical practice, along with reported concerns about ethical sensitivity among professionals, underscores the need for effective ethics training for nursing students to foster accountability, improve dilemma-resolution skills, and ultimately enhance nursing care quality.

In response, many nursing programs have integrated ethics into their core curricula. Research exploring educational interventions to enhance nursing students’ ethical sensitivity has employed diverse strategies, including debate-based teaching, role-playing, gamification, simulation, and flipped classrooms [8–13]. Despite their pedagogical value, these innovative approaches yield inconsistent results. For example, debate-based methods fail to reliably enhance ethical sensitivity [9], and gains from short courses (e.g., 4–8 weeks) are often transient. In contrast, problem-based learning (PBL) and reflective practice demonstrate sustained efficacy in follow-up assessments [14]. Furthermore, significant heterogeneity across studies regarding sample sizes, student backgrounds, and educational stages further complicates the interpretation and comparison of findings.

This inconsistency necessitates a systematic evaluation of the evidence. Previous reviews have provided valuable insights but have limitations relevant to this specific question. Shadi et al. (2024) systematically analyzed 38 studies, clarifying the attributes, antecedents, and consequences of ethical sensitivity and emphasizing the role of ethical knowledge in educational settings [15]. However, their focus was conceptual, and they did not quantitatively assess intervention effectiveness. Spekkink and Jacobs (2021), in a scoping review of 10 studies, proposed a dimensional framework for ethical sensitivity but could not draw definitive conclusions about specific teaching approaches due to methodological diversity [16]. Stolt et al. (2018) systematically reviewed 23 studies on ethics interventions for healthcare professionals and students [17]. However, the analytical complexity and low quality of several studies compromised the rigor of their findings. Tanaka and Tezuka (2022) identified five effective blended pedagogical approaches for developing nursing students’ ethical competencies through a scoping review of 14 quasi-experimental studies [18]. However, they did not examine non-instructional curricular elements.

In summary, existing research on ethical sensitivity in nursing has predominantly emphasized conceptual analysis and scoping specific teaching methods, often focusing on nurses or medical students. Systematic reviews specifically evaluating educational interventions aimed at enhancing ethical sensitivity among nursing students are scarce. Given the pivotal future roles of nursing students and the complex ethical challenges they will encounter, developing and implementing effective educational strategies to strengthen their ethical sensitivity are imperative. Therefore, this systematic review aims to evaluate the efficacy and feasibility of existing educational interventions designed to improve nursing students’ ethical sensitivity, providing an evidence base to inform nursing education practices.

## Methods

### Design

We conducted a systematic review, adhering to the Preferred Reporting Items for Systematic Reviews and Meta-Analyses (PRISMA) checklist. A systematic search was conducted across Chinese and English databases, with stringent inclusion and exclusion criteria for literature selection.

### Search strategy

We conducted a comprehensive and systematic literature search across multiple electronic databases, from the establishment of each database to December 2024. The English-language databases included PubMed, Embase, Medical Literature Analysis and Retrieval System Online (MEDLINE), Web of Science, Cochrane Library, and Cumulated Index in Nursing and Allied Health Literature (CINAHL). The Chinese-language databases included China National Knowledge Infrastructure (CNKI), VIP (Chinese Science and Technology Journal Database), and Wanfang Data.

The search strategy focused on three key aspects. First, for the student population, the following keywords were used: “nursing students,” “undergraduate nursing students,” and “medical students.” Second, concerning educational intervention measures, the keywords included “nursing education,” “educational intervention,” “ethical education,” “ethical training,” “ethical planning,” “teaching strategies,” “learning methods,” “problem-based learning (PBL),” “simulated problem-based learning (SPBL),” and “nursing curriculum.” Finally, concerning morality and ethical sensitivity, the keywords used were “moral sensitivity,” “moral consciousness,” “moral issue sensitivity,” “ethical sensitivity,” “ethical issue sensitivity,” “ethical consciousness,” and “ethical perception” (In this paper, “ethical sensitivity” and “moral sensitivity” are used interchangeably, with “ethical sensitivity” being the preferred term).

Additionally, we employed a manual retrieval method. By carefully reviewing the reference lists of the identified studies and relevant review articles, we identified additional studies that may have been missed during the initial electronic search.

### Inclusion and exclusion criteria

Studies were included if they met the following inclusion criteria: (1) educational interventions aimed at undergraduate nursing students were described and evaluated, (2) measurement results regarding moral or ethical sensitivity were provided, (3) the study design was a randomized controlled trial (RCT) or a quasi-experimental design, (4) the full text was available, (5) the publication was limited to English or Chinese, and (6) the publication date was before December 31, 2024.

The following categories were excluded: (1) research proposals, editorials, conference abstracts, conference proceedings, paper abstracts, and theses; (2) research on educational interventions for graduate nursing students or in-service nurses; and (3) studies without explicitly stated educational methods.

### Quality assessment

Two authors independently evaluated the quality of the studies using the Joanna Briggs Institute critical appraisal tools for RCTs and quasi-experimental studies [19]. Each checklist item was evaluated as “yes,” “no,” “unclear,” or “not applicable.” Discrepancies were resolved through team discussions until consensus was reached. Supplement 1 provides an overview of the quality of the studies.

### Data extraction and synthesis

Two authors independently extracted relevant data from the included studies and organized them into an Excel spreadsheet. This provided a detailed summary of the key features of the included studies, including author, publication year, study design, sample characteristics, study setting, study country, specific details of the intervention measures, measuring tools, and measurement outcomes. To ensure data accuracy and reliability, the authors held in-depth discussions to resolve discrepancies in the extracted data. When interpretations differed, all authors collaboratively analyzed the data and reached a consensus.

Given the significant heterogeneity of the included studies regarding intervention methods, assessment tools, and cultural contexts, a meta-analysis was not feasible. Instead, a systematic review with a narrative synthesis approach was adopted. The interventions and outcomes were systematically classified into predefined categories based on the extracted data. The findings within each category were then summarized and interpreted descriptively.

## Results

### Selection process

**Fig. 1:**
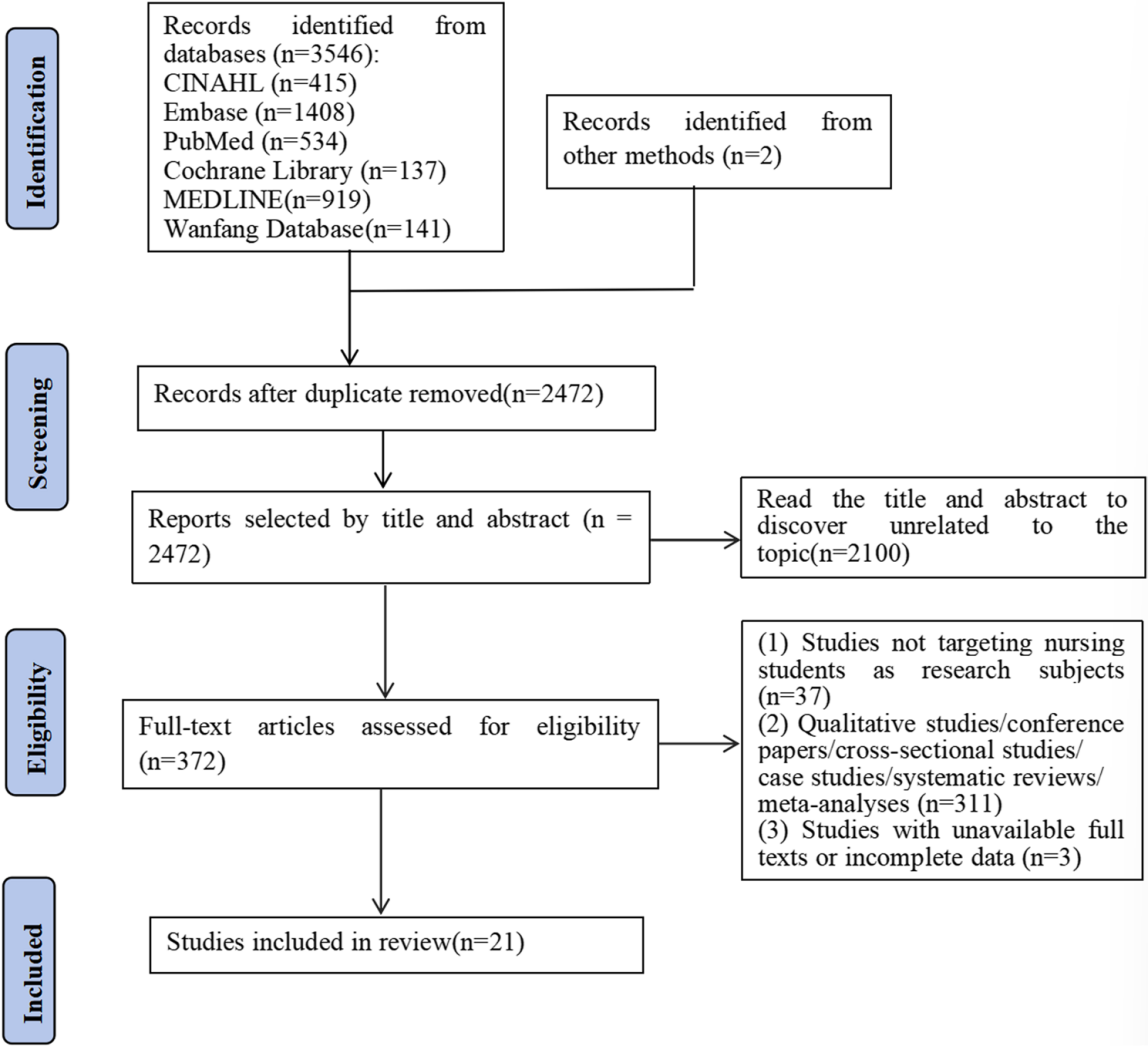
PRISMA flowchart showing the article selection process

Initially, a search in the databases yielded 3,546 records. Through manual screening of relevant reviews and reference lists of included articles, an additional 2 records were identified, bringing the total to 3,548 records. After removing 1,076 duplicate records, 2,472 records remained and entered the title and abstract screening phase. Two reviewers independently screened these records and excluded 2,100 records that were irrelevant to the topic. This resulted in 372 records being selected for full-text retrieval. After full-text review, 351 records were excluded for specific reasons: 37 studies did not focus on nursing students as the research subjects; 311 were ineligible study types, such as qualitative studies, conference papers, cross-sectional studies, case studies, systematic reviews, or meta-analyses; and 3 were excluded due to unavailable full texts or incomplete data. Ultimately, 21 studies met all eligibility criteria and were included in the systematic review. The complete selection process is detailed in the PRISMA flow diagram (see Fig. 1).

### Study characteristics

As shown in Table 1, this review includes 21 studies published between 2015 and 2024, with sample sizes ranging from 30 to 344 participants. In terms of research design, the majority of the studies (*n* = 16) adopted quasi-experimental design [8–13, 20–28]. In addition, five studies were designed as randomized controlled trials [1, 14, 29–31]. In terms of geographical distribution, these studies were conducted in various countries. Seven studies were from Turkey [1, 22, 24, 25, 27, 29, 30], six from Iran [10, 13, 14, 23, 26], and four studies in mainland China [7, 8, 12, 31]. South Korea conducted two studies [9, 20], while other regions including Taiwan [21], India [11], and Malaysia [28] each contributed one study.

**Table 1:** General characteristics of the included studies (*n* = 21)

First Author (Year)	Country	Study Design Type	Study Participants	Sample Size
Yeom et al., 2017 [20]	Korea	quasi-experimental	senior nursing undergraduate students	*N* = 70
Lee et al., 2017 [21]	Taiwan	quasi-experimental	third-year student nurses in a five-year junior college nursing program	*N* = 234
Zia et al., 2023 [14]	Iran	RCT	Undergraduate nursing students in their 5th and 7th semesters	*N* = 74 (PBL group = 25; Reflective group = 25; Control group = 24)
Maddineshat et al., 2019[11]	Indian	quasi-experimental	Fourth-semester undergraduate nursing students	*N* = 30
Baykara et al., 2015[1]	Turkey	RCT	Fourth-year nursing students	*N* = 50 (intervention group = 25; control group = 25)
Qu et al., 2024 [7]	China	quasi-experimental	undergraduate nursing students	*N* = 161 (intervention group = 88; control group = 73)
Yüksel Kaçan., 2022 [22]	Turkey	quasi-experimental	Second-year nursing students	*N* = 100 (Intervention group = 36; Control group = 64)
Ertuğrul et al., 2022 [29]	Turkey	RCT	Second-year nursing students	*N* = 100 (Intervention group = 50; Control group = 50)
Kim & Park, 2019[9]	Korea	quasi-experimental	Senior nursing students	*N* = 64 (debate group = 35, lecture group = 29)
Azarkish et al., 2023[13]	Iran	quasi-experimental	First-, second-, and third-year nursing students	*N* = 120 (flipped classroom group = 40; SMS intervention group = 40; control group = 40)
Jasemi et al., 2020 [23]	Iran	quasi-experimental	Sixth- and eighth-semester nursing students	*N* = 83 (intervention group = 42; control group = 41)
Kucukkelepce et al., 2020 [24]	Turkey	quasi-experimental	nursing students	*N* = 89 (intervention group = 45; control group = 44)
Ziyai et al., 2024 [25]	Turkey	quasi-experimental	Second-year nursing students	*N* = 145
Ekramifar et al., 2018[26]	Iran	quasi-experimental	Fourth-semester nursing students	*N* = 70(intervention group = 35; control group = 35)
Nesime & Belgin, 2022[30]	Turkey	RCT	nursing students	*N* = 80(intervention group = 40; control group = 40)
Jasemi et al., 2022 [10]	Iran	quasi-experimental	Sixth-, seventh-, and eighth-semester nursing students	*N* = 114(role-playing group = 38; lecture group = 38; control group = 38)
Yang et al., 2024 [31]	China	RCT	Second-year nursing students	*N* = 344 (intervention group = 170; control group = 174)
Zhang et al., 2024 [12]	China	quasi-experimental	nursing students	*N* = 41
Luo et al., 2024 [8]	China	quasi-experimental	Fourth-year nursing students	*N* = 120(intervention group = 60; control group = 60)
Su et al., 2024 [27]	Turkey	quasi-experimental	Third-year nursing students	*N* = 44
Tang & Yahya, 2024 [28]	Malaysia	quasi-experimental	First-, second-, and third-year undergraduate nursing students	*N* = 55

*RCT *Randomized Controlled Trial, *PBL *Problem-Based Learning, *SPBL *Simulation Problem-Based Learning, *SMS *Short Message Service

### Classification and analysis of ethical education interventions

The narrative analysis of the 21 included studies revealed that the educational interventions could be meaningfully classified into a two-dimensional framework focusing on two core dimensions: teaching methods and teaching content. The former includes two main categories: teacher-centered didactic teaching and student-centered situational participatory teaching. The latter includes three categories: basic theory, focusing on ethical principles and professional standards; clinical practice, emphasizing the practical application of ethical decision-making; and interdisciplinary integration, highlighting the importance of multi-professional collaboration and comprehensive application. The detailed characteristics of interventions across all 21 included studies are summarized in Table 2.

**Table 2:** Educational interventions in the included studies (*n* = 21)

First author (Year)	Intervention for the experimental group	Intervention for the control group		Intervention duration	Follow-up time	Measuring tools	Main outcomes
Yeom et al., 2017[20]	The educational content included topics such as nursing ethical principles, ethical decision-making processes, nursing professionalism, and bioethical issues. The teaching methods included traditional lectures, group discussions, question-and-answer sessions, and watching ethics-related films.	None		7 weeks	/	K-MSQ	The total moral sensitivity score did not change before and after the intervention.
Lee et al., 2017 [21]	Three teaching strategies employing visual, auditory, and kinesthetic approaches were implemented. The curriculum included analyzing *The Diary of Florence Nightingale*, examining kidney transplant patient narratives, and applying the four-topic method to clinical ethics scenarios.	None		12 weeks	/	MMSQ-SN	Moral sensitivity improved significantly after the intervention.
Zia et al., 2023 [14]	Two groups were created. The problem-based learning (PBL) group engaged in group discussions on ethical dilemma scenarios, covering nursing professional ethics, nursing ethical codes, patient rights, ethical decision-making, and professional communication. The reflective practice group covered the same content as the previous group but with a reflective practice approach based on Atkins and Murphy’s theory, focusing on discussions and reflections.	Conventional nursing education courses		4 weeks	3 months	MSQ	The total moral sensitivity scores were significantly higher in the PBL and reflective practice groups than in the control group immediately after the intervention and at follow-up.
Maddineshat et al., 2019[11]	A game-based ethics teaching approach was employed, integrating problem-solving and gameplay. Twelve types of games were employed (such as card sorting, role-playing, drawing, or artistic creation) and incorporated into the new curriculum in Iranian nursing programs.	None		17 weeks	/	MSQ	The intervention group had significantly higher total moral sensitivity scores than the control group.
Baykara et al., 2015[1]	The content covered ethical principles, patient rights, nursing ethical standards, characteristics of ethical issues, medical accidents, and medication administration errors. The teaching methods included lectures, question-and-answer sessions, discussions, and case studies.	Conventional nursing education courses		4 weeks	/	MSQ	No significant difference in moral sensitivity was observed between the intervention and control groups.
Qu et al., 2024 [7]	The content covered nursing interpersonal relationships, ethics in clinical practice, and ethics in public health services. Students engaged in problem-oriented learning; answered questions; participated in group discussions role-playing activities; and conducted simulation practices.	PBL		8 weeks	/	MSQ-CV	Moral sensitivity scores were significantly higher in the SPBL group than in the PBL group.
Yüksel Kaçan., 2022[22]	The course covered topics such as health and culture, transcultural nursing, cross-cultural communication, cultural sensitivity, and nursing practices across religions. Interactive teaching methods such as lectures, case analysis, group discussions, role-playing, watching films, and reading articles were employed.	Conventional nursing education courses		14 weeks	/	MSQ	Students in the transcultural nursing course had significantly higher moral sensitivity than those in the control group.
Ertuğrul et al., 2022[29]	The ethics laboratory program included interactive teaching methods such as ethical scenarios, case studies, role-playing, group discussions, project papers, and watching films.	Conventional nursing education courses		8 weeks	/	MSC	No significant difference in moral sensitivity was observed between the intervention and control groups.
Kim & Park, 2019[9]	Through 16 rounds of debates (affirmative and negative sides), each session included segments such as introduction, cross-examination, rebuttal, conclusion, and wrap-up, revolving around 10 ethical dilemma cases, including human cloning, conflicts between confidentiality and nursing duties, and meaningless life-sustaining care.	Lecture-based teaching		8 weeks	/	K-MSQ	No statistically significant difference in moral sensitivity was observed between the debate and lecture groups.
Azarkish et al., 2023[13]	Two groups were created. The flipped classroom group engaged in activities such as concept map drawing, group discussions, and role-playing. The SMS service group received ethical education-related messages, sent via WhatsApp.	Conventional nursing education courses		1 month	1 month	MSQ	Both flipped classroom and SMS service significantly improved students’ moral sensitivity.
Jasemi et al., 2020[23]	Students received lecture-based instruction on the nursing code of ethics, covering basic nursing ethics concepts, the importance of ethics, and ethical codes in nursing.	Conventional nursing education courses		6 h	2 months	MSQ	The lecture group had significantly higher moral sensitivity than the control group.
Kucukkelepce et al., 2020 [24]	Ethics education involved standardized patients, with students engaging in face-to-face interviews with the patients, followed by group discussions and feedback sessions.	Case analysis		3 weeks	/	MSQ	The standardized patient group had significantly improved moral sensitivity compared to the case analysis group.
Ziyai et al., 2024 [25]	The flipped classroom and jigsaw learning models were used for ethics education. Students acquired foundational knowledge by watching videos before class, followed by interactive learning through group discussions and case analysis.	None		5 weeks	/	MMSQ-SN	No significant difference in moral sensitivity was observed between the intervention and control groups.
Ekramifar et al., 2018 [26]	The course content included spiritual concepts, spiritual skills, the role of spirituality in education and clinical practice, self-awareness, the relationship between spirituality and mental health, and religious–spiritual conflicts.	Conventional nursing education		6 weeks	1 month	K-MSQ	Spiritual training significantly improved students’ moral sensitivity.
Nesime&Belgin,2022 [30]	The control group received an advocacy education curriculum based on Bloom’s Taxonomy of Educational Objectives, covering topics such as health inequality, social justice, and laws and regulations, as well as practical activities including poster creation and online support work.	Conventional nursing education courses		11 weeks	/	MSQ	Advocacy education significantly enhanced students’ moral sensitivity.
Jasemi et al., 2020[23]	Two groups were created. The role-playing group engaged in scenario simulation, role-playing, and mock trials. In the lecture group, the teacher explained ethical theories and cases in class.	Conventional nursing education courses		2 weeks	2 months	MSQ	The role-playing group had significantly higher moral sensitivity scores than the lecture group, both immediately after the intervention and two months later.
Yang et al., 2024 [31]	Combining case-based teaching, students were immersed in real-life ethical scenarios through methods such as scenario imagination, role-playing, and mock trials to analyze and make decisions on ethical issues.	Conventional case-based teaching method		18 weeks	/	MSQ-NS	The intervention group had significantly better ethical sensitivity than the control group.
Zhang et al., 2024[12]	The curriculum combined theoretical and experiential learning. The former included nursing ethics fundamentals, principles, professional relationships, and bioethical issues. The latter involved clinical ethics observation and simulated scenarios, with students analyzing real-world cases, participating in guided ethical reflections, and receiving instructor feedback.	None		4 weeks	/	ESQ-NS	The total ethical sensitivity score was significantly higher after the intervention than before it.
Luo et al., 2024 [8]	Debate-based ethics teaching was performed, including selecting ethical cases, determining debate topics, organizing students for pre-class ethical learning, explaining medical ethics guidelines and cases, introducing the debate-based ethics teaching model, and organizing students for debate.	Traditional nursing ethics teaching model		6 months	/	ESQ-NS	The intervention group had significantly better ethical sensitivity than the control group.
Su et al., 2024 [27]	The nursing ethics education course covered topics such as the historical development of ethics and ethical concepts, theories, principles, and practice. The teaching methods included lectures, question-and-answer sessions, case discussions, and video analysis.	None		14 weeks	/	MSQ	The total ethical sensitivity score was significantly higher after the intervention than before it.
Tang & Yahya, 2024 [28]	Real-life clinical scenarios were simulated through the analysis of six cases, covering themes such as respect for individuals, fair distribution, and patient privacy. The definition of ethical dilemmas and moral sensitivity, as well as Kohlberg’s theory of moral development and biomedical ethics principles, were also included.	None		2 months	/	ESQ-NS	Students’ moral sensitivity improved significantly after the intervention.

*K-MSQ *The Korean version of the Moral Sensitivity Questionnaire *MMSQ-SN *Modified Moral Sensitivity Questionnaire for Student Nurses, *MSQ *The Moral Sensitivity Questionnaire, *MSQ-CV *The Chinese version of the Moral Sensitivity Questionnaire, *MSC *Moral Sensitivity Scale, *MSQ-NS *Moral Sensitivity Questionnaire for Nursing Students, *ESQ-NS *Ethical Sensitivity Questionnaire for Nursing Students

### Teaching methods

#### Analysis of the included studies indicated that the teaching methods adopted fell into two distinct categories

##### Teacher-centered didactic teaching

This approach constructs a comprehensive knowledge system through systematic curriculum design and theoretical instruction, often followed by case analysis for theory application. For instance, Jasemi et al. (2020) organized a three-day lecture series that systematically covered five core areas of nursing ethics in Iran, as well as clinical application guidelines [23]. Similarly, Yeom et al. (2017) adopted a traditional teaching model focused on theoretical lectures and supplemented by case discussions and film analysis [20]. Other examples include Baykara et al. (2015), who designed a two-stage training program involving systematic explanation of ethical principles followed by case analysis [1], and Ekramifar et al. (2018) and Su et al. (2024), who employed a similar combination of theoretical instruction and case analysis [26, 27]

##### Student-centered situational participatory teaching

This method, grounded in constructivist theory, fosters students’ self-directed learning and reflective skills through interactive methods in real or simulated contexts. The innovations in this category were evident in three aspects specific techniques, teaching models, and blended learning. Regarding specific techniques, studies demonstrated effectiveness through role-playing [10], a multimodal teaching program based on the VAK learning model [21], an improved seven-step PBL process [14], and the integration of role-playing into PBL (SPBL) [7]. The use of standardized patients with feedback [24] and focused group case discussions on clinical situations [28] also showed positive results. In terms of teaching models, researchers developed comprehensive methods involving interactive activities [11], a three-stage trial-role debate using concept mapping [31], and a progressive approach combining theory, debate, and reflection [9]. Several studies explored various forms of clinical situational simulation teaching [8, 22, 29]. Furthermore, significant progress was made in blended teaching models, which combined strategies such as flipped classrooms with online assessments [13], integrated flipped classrooms with jigsaw learning [25], established three-dimensional models combining theory, situational participation, and online extension [30], and demonstrated long-term effectiveness through the integration of classroom instruction, clinical observations, and practice [12].

### Teaching content

#### The analysis of educational content across the studies led to the development of a three-dimensional framework

##### Basic theory

This dimension encompasses the core conceptual framework of nursing ethics, including professional ethical principles, behavioral norms, and ethical decision-making models. Several studies contributed to defining this dimension. Jasemi et al. (2020) proposed a five-dimensional framework based on national regulations [23], while Su et al. (2024) systematically organized ethical theories and principles like deontology and the four principles of medical ethics [27]. Yeom et al. (2017) examined the foundations of ethical norms and decision-making models [20], Nesime and Belgin (2022) applied Bloom’s Taxonomy to construct an educational objectives model [30], and Yang et al. (2024) described a comprehensive four-level theoretical framework [31].

##### Clinical practice

This dimension emphasizes the practical application of ethical decision-making in healthcare settings. Studies focusing on this area explored a range of clinical ethical issues. Baykara et al. (2015) focused on patients’ rights, informed consent, and privacy protection [1]. Ertuğrul et al. (2022) investigated ethical challenges in special situations like patient safety and end-of-life care [29]. Kucukkelepce et al. (2020) covered ethical norms, patient rights, and clinical decision-making disputes [24]. Other studies analyzed conflicts from an ethical perspective, highlighting fairness [8], developed analysis models based on typical clinical situations [28], and systematically categorized common clinical dilemmas [11].

##### Interdisciplinary integration

This dimension highlights the importance of multi-professional collaboration and explores the intersection of nursing ethics with other fields. Recent research has expanded into interdisciplinary areas. Yüksel Kaçan (2022) constructed a framework for cross-cultural nursing ethics, exploring cultural sensitivity and diverse value systems [22]. Ekramifar et al. (2018) integrated spiritual belief theory, focusing on spiritual needs and care [26]. Kim and Park (2019) analyzed ethical dilemmas associated with emerging biotechnologies [9], and Su et al. (2024) examined ethical challenges from assisted reproductive technologies [27].

## Discussion

Current nursing ethics education faces critical challenges, including the need to innovate beyond traditional teaching methodologies, address cross-cultural ethical conflicts in globalized healthcare, and navigate ethical dilemmas arising from digital health technologies. In specialized domains, such as spiritual care, existing approaches remain inadequate for cultivating students’ ethical decision-making and practical competencies.

The traditional lecture method plays a fundamental role in nursing ethics education, especially in terms of ethical norms and policy frameworks [32–34]. However, its effectiveness in cultivating ethical sensitivity among nursing students varies. For example, Jasemi et al. (2020) demonstrated that lectures can enhance students’ ethical sensitivity [23]. Similarly, Ekramifar et al. (2018) and Su et al. (2024) reported that ethical sensitivity improved through a combination of theoretical guidance and case analysis, as along with diverse teaching methods [26, 27]. In contrast, Yeom et al. (2017) and Baykara et al. (2015) found that the unidirectional transmission model has limitations in cultivating ethical practice skills [1, 20]. These differences indicate that the effectiveness of ethical competence development is closely related to the depth of interaction in teaching. Passive learning, characterized by a lack of interaction, fails to engage students in deep reflection on complex ethical issues [20] and limits the sustainability of teaching outcomes [35].

Building on the need for more interactive approaches, situational participatory teaching involves the creation of a highly simulated clinical ethics situation for students through interactive methods such as role play and scenario simulation. Among the 21 studies included in this review, 16 employed situational participatory teaching, with 13 of these demonstrating significant improvement in students’ ethical sensitivity. Qu et al. (2024) found that this method not only enhances students’ ability to identify ethical issues but also promotes the development of critical thinking and teamwork skills [7]. Zia et al. (2023) noted that structured case analysis helps students transform abstract ethical principles into clinical decision-making capabilities more effectively [14]. Thus, multiple studies [24, 25] have shown that diversified teaching strategies integrating specific cases, teaching aids, and scenario simulations are more effective than single teaching methods. However, the implementation of this approach faces challenges, such as the need for substantial resources and high-level teaching staff. Moreover, the singularity of existing teaching scenarios limits students’ understanding of complex ethical issues. For example, Hu et al. (2024) found that when teaching scenarios focus solely on single-patient care, students often fail to grasp the ethical dimensions of medical resource allocation and may even misjudge unfair resource distribution as a non-ethical issue [36].

To address these limitations, technological innovations such as virtual reality (VR) have shown significant progress in nursing ethics education. In skills training, simulated clinical environments constructed using VR allows nursing students to safely practice procedures such as venipuncture, wound care, and cardiopulmonary resuscitation. This method improves technical accuracy and clinical adaptability [37]. In ethical decision-making training, VR significantly enhances the practicality of nursing decisions and depth of reflection by simulating scenarios and providing dynamic interactions. In scenarios involving privacy protection and informed consent, VR provides real-time feedback on the consequences of violations, helping students understand the relationship between confidentiality and respect for autonomy [38]. For complex decisions, such as end-of-life care, VR uses branching narratives and data visualization to demonstrate the impact of different choices, helping students understand the balance between beneficence and non-maleficence [39]. This simulation helps cultivate critical thinking and improves clinical ethical decision-making skills. Future research should further explore specific implementation plans for technology-empowered education, such as modular curriculum design, faculty training programs, and cost-benefit analysis, to facilitate the transformation from theory to practice.

Beyond teaching methodologies, the evolving healthcare landscape introduces new ethical dimensions that curricula must address. Currently, nursing ethics research covers areas such as cross-cultural nursing ethics [22], spiritual care ethics [26], bioethics [9], and the ethics of assisted reproductive technology [27]. However, with the rapid development of medical technology, nursing practice faces many new ethical challenges. Obermeyer et al. (2019) found that a certain AI system, owing to biased training data, significantly underestimated health risks for Black patients compared to White patients, thereby affecting the fairness of medical resource allocation [2]. Similarly, Google’s DeepMind, which used patient data without sufficient informed consent, exposed ethical deficiencies in medical data governance [3]. Additionally, AI medical devices may weaken patient autonomy. For example, patients with diabetes using AI-controlled insulin pumps may face an increased risk of hypoglycemia if the system cannot adjust insulin dosage based on physical activity levels [40]. Although the World Health Organization (WHO) has issued ethical guidelines for AI in healthcare to emphasize the integration of technological ethics into nursing education, existing nursing curricula are still insufficient in this regard [41].

Parallel to technological challenges, public health crises have highlighted decision-making dilemmas faced by nursing professionals in ethical conflicts between the “individual” and “community.” Research showed that nurses were often required to make difficult trade-offs between individual treatment rights and public health interests [42]. Furthermore, while treating patients, they were required to choose between “protecting their own safety” and “fulfilling their professional duties” [43]. Additionally, during epidemic tracking and information disclosure, nurses had to balance “protecting patient privacy” with “public health transparency” [44]. Such practical challenges highlight the urgent need to integrate public health ethics into nursing curricula. It is also important to understand the complex relationship between students’ bioethical awareness and ethical perceptions of AI. Yang (2024) found that higher levels of bioethical awareness may lead to excessive caution toward AI applications, reducing technology acceptance intentions, while individuals with lower ethical sensitivity may overlook potential risks [45]. This cognitive difference highlights the importance of integrated ethics education.

To address emerging ethical challenges effectively, establishing a multi-tiered AI ethics competency training framework for nursing students is essential. First, core issues related to AI should be systematically integrated into nursing curricula. By employing case analysis combined with clinical AI application scenarios, students’ ethical critical thinking can be developed [46]. Second, students’ abilities to identify technological risks, particularly in AI ethics, should be enhanced through digital literacy training and ethical sensitivity exercises. Finally, in public health ethics education, a crisis decision-making sandbox that incorporates cross-cultural review mechanisms should be employed to develop students’ dialectical capacity to balance individual rights, collective welfare, and cultural differences during emergencies.

The incorporation of cross-cultural review mechanisms underscores the broader need for cultural adaptability in nursing ethics education. The current nursing ethics education system faces significant challenges related to cultural adaptability. Existing curricula are largely designed from a Western cultural perspective, with teaching cases that struggle to address the conflicts arising from diverse value systems in globalized nursing practices (ICN, 2019) [47]. Yüksel Kaçan (2022) proposed a cross-cultural curriculum framework covering core themes such as the relationship between health and culture, cultural sensitivity, and religious customs [22]. However, only one study in the literature has explored cross-cultural courses, and its application has been limited to specific cultural contexts, such as Turkey. With the acceleration of globalization and increased international migration, nursing professionals urgently need to develop cross-cultural ethical competencies to thoroughly understand patients’ cultural backgrounds and provide personalized care that meets their cultural needs [48].

Research indicates a significant positive correlation between cross-cultural competence and ethical sensitivity in nursing students [49]. Cultural differences in ethical perception are primarily reflected in divergent value orientations. Western culture emphasizes individualism and focuses on individual rights and needs, while Eastern culture leans toward collectivism, prioritizing social harmony and group interests [41]. This fundamental difference requires nursing ethics education to integrate multicultural perspectives and avoid racial centrism. In the Middle East, nursing ethics education emphasizes integration with Islamic culture, implementing “modesty care protocols” based on religious doctrines to regulate interactions between healthcare providers and patients of the opposite sex [50]. Additionally, an ethics consultation mechanism involving religious scholars provides professional guidance for potentially conflicting clinical decisions [51]. In contrast, nursing practices in East Asia exhibit family-centered cultural characteristics. In clinical practice, a stepwise diagnostic disclosure strategy is adopted, with priority given to negotiating treatment plans with patients’ families [52]. The ethical mediation ability of healthcare professionals is improved through structured collective decision-making workshops [53].

Given the impact of cultural differences on ethical decision-making in nursing, educational institutions should tailor cross-cultural curricula to the cultural characteristics and ethical norms of target countries. At the practical teaching level, the development of immersive scenario simulation training based on VR technology is recommended. By recreating cross-cultural conflict scenarios, students’ cultural sensitivity and communication skills can be cultivated. During clinical internships, structured cultural reflection logs, based on models such as Gibbs’ reflection cycle, can be used to guide students in recording and analyzing cross-cultural events to deepen their understanding of cross-cultural nursing ethics.

Closely linked to cultural competence is the dimension of spiritual care, which is fundamental to holistic nursing practice. Holistic nursing, which has been established as a core practice requirement for registered nurses and as an international standard for nursing practice [54], is fundamentally committed to promoting the overall health of all individuals. This model emphasizes the importance of meeting the multidimensional health needs of patients, with spiritual care recognized as an essential component [55]. With the development of global health concepts, the strategic importance of spiritual care has become increasingly important. Both the WHO (WHO, 2018) and the United Nations have designated palliative care, which includes a spiritual dimension, as an ethical obligation for healthcare systems [56]. The International Council of Nurses’ Code of Ethics (2012) explicitly stipulates that nurses should respect and meet patients’ spiritual needs [57]. In addition, the practice standards of both the Joint Commission and the Quality and Safety Education for Nurses (QSEN) require the extension of the “patient-centered” care philosophy to the spiritual domain [58, 59]. These policy orientations have prompted countries to incorporate spiritual care into the medical education accreditation system, thereby imposing new requirements on nursing education.

Although the value of spiritual care is widely recognized in academia, research in this field remains insufficient, and the development of nursing education is severely limited. Existing studies indicate that nurses generally lack the confidence and ability to provide spiritual care due to inadequate education and training, ambiguous clinical roles, and a limited understanding of the concept of spirituality [60]. Karaman et al. (2022) found that the low coverage of spiritual care courses in undergraduate nursing education results in graduates being unable to meet patients’ spiritual needs [61]. Additionally, cultural and religious diversity complicates spiritual care, while the scarcity of research outside Western contexts makes it difficult to apply existing educational models globally [62]. Notably, among the 21 included studies, only one specifically explored the efficacy of spiritual care training programs,31 revealing that research in this area remains limited.

To address these gaps, nursing curriculum reforms should adopt a multifaceted approach. At the curricular level, this requires establishing a dedicated core module on spiritual care to cultivate students’ abilities in spiritual needs assessments and clinical interventions. Furthermore, a longitudinal integration strategy must embed relevant content throughout the educational continuum, from foundational theory to clinical practice. Crucially, cultural adaptability principles must be incorporated, encompassing diverse perspectives such as Buddhist views on life and death in East Asian contexts and Islamic medical ethics relevant to Middle Eastern populations [63, 64]. Regarding pedagogy, a combined experiential and reflective approach is recommended. Case-based clinical reasoning exercises should provide authentic learning opportunities, while standardized patient encounters should facilitate the development of cross-cultural communication skills. These methods can be enhanced through high-fidelity simulation scenarios that create realistic training environments. Crucially, all experiential components should be reinforced through structured reflective practice using established frameworks such as Gibbs’ reflection cycle [65].

Ultimately, the development of ethical sensitivity requires a comprehensive educational system that integrates the pedagogical, technological, cultural, and spiritual dimensions discussed above. Current research indicates that the development of ethical sensitivity among nursing students is a complex phenomenon. Cevheroğlu (2024) found that students during their clinical internships demonstrated significantly higher ethical sensitivity than their pre-clinical counterparts [66]. However, Kim and Joung (2019) reached the opposite conclusion [67]. This contradiction may stem from defects in current ethics education, as moral education mainly focuses on the theoretical learning stage, while situational factors in clinical practice may lead to students’ cognitive biases regarding ethical issues. Given the significant positive correlation between ethical sensitivity and ethical behavior [68], the establishment of a comprehensive education system that bridges theoretical learning and clinical practice is urgently needed. However, a significant gap remains in current educational practices. Although this study included nursing students from all stages, the educational intervention mainly focused on theoretical learning in schools and has not been fully extended to clinical practice.

Research has confirmed that ethical interventions are highly effective among nurses. For example, the poetry-based ethics education developed by Rashidi et al. (2022) has been found to effectively improve nurses’ ethical reflection abilities through artistic expression [69], while the reminiscence training adopted by Bahrieni et al. (2022) has been found to strengthen situational adaptability by sharing clinical experiences through narrative storytelling [70]. However, these interventions often fail to optimize the learning characteristics and clinical needs of nursing students during internships. Moreover, the fragmented implementation of short-term projects makes it difficult to achieve sustained improvements in ethical sensitivity [23]. This highlights the need to construct a comprehensive ethics education system that integrates collaboration between schools and clinical settings.

Wocial (2010) emphasized that ethics education should adopt a high-frequency, contextualized training model to facilitate the internalization of knowledge [71]. Research has shown that interactive and participatory teaching methods not only enhance students’ understanding and retention of ethical principles but also strengthen moral commitment [72]. Educators should stimulate students’ reflection and create a classroom atmosphere that encourages exploration through heuristic questioning and case discussion. Furthermore, the development of collaborative university-hospital partnerships is critical. Such partnerships can draw on successful principles of integrated training-such as combining self-regulation, ethical sensitivity, and insight development-tailoring them to local contexts to support nursing students throughout their educational journey [73]. Finally, in the clinical education phase, contextualized learning designs should be emphasized. Holland (1999) confirmed that experiential teaching methods, such as simulated scenarios and role-playing, are more effective than traditional lectures in promoting moral development [74]. Therefore, clinical internships should provide sufficient opportunities to simulate decision-making and help students internalize ethical principles through scenario reproduction.

### Limitations

Although this study systematically organized and analyzed educational interventions for nursing students’ ethical sensitivity, several limitations should be acknowledged. First, significant heterogeneity was observed across the included studies regarding intervention duration, sample size, and evaluation tools, which precluded a meta-analysis of the results. Second, the generalizability of the findings is limited by the geographical concentration of studies in specific regions, notably Iran and Turkey. The lack of studies from Western European and other Western contexts may reflect differing regional research priorities, such as a focus on qualitative methodologies or curriculum evaluations, rather than the quantitative experimental designs required for this review. Third, most studies assessed only short-term effects, lacking long-term follow-up to evaluate the sustainability of interventions. Fourth, methodological limitations, including the predominant use of quasi-experimental designs and self-reported measures, may have introduced measurement bias. Finally, the analysis lacks depth in addressing complex and emerging ethical domains, such as AI ethics and cross-cultural nursing.

Future research should employ standardized measures, diversify samples geographically and culturally, and incorporate longitudinal designs to better evaluate the long-term impact and sustainability of interventions. Broader search strategies in future reviews could also help capture a wider spectrum of research methodologies and geographical contexts.

## Conclusion

This study systematically reviewed 21 relevant studies and demonstrated that situational teaching methods generally enhance ethical sensitivity among nursing students, whereas traditional lecture-based approaches yield limited effects. However, due to methodological heterogeneity across the included studies and the absence of a meta-analysis, these findings still require further validation through high-quality research.

Building on these insights, it is recommended that contemporary nursing ethics education adopt more interactive and participatory strategies-such as situational teaching-to strengthen students’ practical engagement and ethical reasoning skills. Furthermore, nursing curricula should be updated to incorporate emerging ethical topics, including AI ethics, public health ethics, and transcultural nursing, in order to broaden students’ knowledge. Finally, educational structures should be optimized to foster a seamless integration of theory and practice, using a full-cycle training model that supports the coordinated development of students’ theoretical understanding and clinical competencies. Together, these measures will better equip future nursing professionals to navigate ethical decision-making in complex healthcare settings.

## Supplementary Information

Supplementary Material 1.

## Data Availability

All data generated or analysed during this study are included in this publishedarticle and its supplementary information files.
