# Supplementary material for: A systematic review of educational interventions to enhance ethical sensitivity in nursing students in Asia and the Middle East
Source: BMC Med Ethics. 2025 Dec 30;26:185. doi: 10.1186/s12910-025-01334-x (PMC12754892; doi:10.1186/s12910-025-01334-x)
Supplement: Supplementary file 1 — Supplementary Material 1. [file 12910_2025_1334_MOESM1_ESM.docx]

**Supplement 1.** Methodological quality assessment results of included studies.

| **First author (year)** | | **1** | **2** | **3** | **4** | **5** | **6** | **7** | **8** | **9** | **10** | **11** | **12** | **13** |
| --- | --- | --- | --- | --- | --- | --- | --- | --- | --- | --- | --- | --- | --- | --- |
| RCT | Zia et al.,2023[14] | Y | U | Y | U | U | U | Y | Y | Y | Y | Y | Y | Y |
|  | Baykara et al., 2015[20] | Y | U | Y | U | U | U | Y | Y | Y | Y | Y | Y | Y |
|  | Ertuğrul et al., 2022[21] | Y | U | Y | U | U | U | Y | Y | Y | Y | Y | Y | Y |
|  | Nesime&Belgin., 2022[22] | Y | Y | Y | N | N | Y | Y | Y | Y | Y | Y | Y | Y |
|  | Yang et al., 2024[23] | Y | U | Y | U | U | U | Y | Y | Y | Y | Y | Y | Y |
|  |  | **①** | **②** | **③** | **④** | **⑤** | **⑥** | **⑦** | **⑧** | **⑨** |  |  |  |  |
| quasi-  experimental | Yeom et al., 2017[24] | Y | Y | Y | Y | Y | Y | Y | Y | Y |  |  |  |  |
|  | Lee et al., 2017[25] | Y | Y | Y | Y | Y | N | Y | Y | Y |  |  |  |  |
|  | Maddineshat et al., 2019[11] | Y | Y | Y | Y | Y | Y | Y | Y | Y |  |  |  |  |
|  | Qu et al., 2024[26] | Y | Y | Y | Y | Y | Y | Y | Y | Y |  |  |  |  |
|  | Yüksel Kaçan.,  2022[27] | Y | Y | Y | Y | Y | N | Y | Y | Y |  |  |  |  |
|  | Kim & Park., 2019[9] | Y | Y | Y | Y | Y | N | Y | Y | Y |  |  |  |  |
|  | Azarkish et al.,  2023[13] | Y | Y | Y | Y | Y | Y | Y | Y | Y |  |  |  |  |
|  | Jasemi et al., 2020[28] | Y | Y | Y | Y | Y | N | Y | Y | Y |  |  |  |  |
|  | Kucukkelepce  et al., 2020[29] | Y | Y | Y | Y | Y | N | Y | Y | Y |  |  |  |  |
|  | Ziyai et al., 2024[30] | Y | U | Y | Y | Y | N | Y | Y | Y |  |  |  |  |
|  | Ekramifar et al.,  2018[31] | Y | Y | Y | Y | Y | Y | Y | Y | Y |  |  |  |  |
|  | Jasemi et al., 2022[10] | Y | Y | Y | Y | Y | N | Y | Y | Y |  |  |  |  |
|  | Zhang et al., 2024  [12] | Y | U | Y | Y | Y | Y | Y | Y | Y |  |  |  |  |
|  | Luo et al., 2024[8] | Y | U | Y | Y | Y | N | Y | Y | Y |  |  |  |  |
|  | Su et al., 2024[32] | Y | U | Y | Y | Y | Y | Y | Y | Y |  |  |  |  |
|  | Tang & Yahya.,  2024[33] | Y | U | Y | Y | Y | Y | Y | Y | Y |  |  |  |  |

Note: RCT: Randomized Controlled Trial; Y:Yes; N: No; U:Unclear

1Was true randomization used for assignment of participants to treatment groups? 2Was allocation to treatment groups concealed? 3Were treatment groups similar at the baseline? 4Were participants blind to treatment assignment? 5Were those delivering the treatment blind to treatment assignment? 6Were treatment groups treated identically other than the intervention of interest? 7Were outcome assessors blind to treatment assignment? 8Were outcomes measured in the same way for treatment groups? 9Were outcomes measured in a reliable way? 10Was follow-up complete and, if not, were differences between groups in terms of their follow-up adequately described and analyzed? 11Were participants analyzed in the groups to which they were randomized? 12Was appropriate statistical analysis used? 13Was the trial design appropriate and any deviations from the standard RCT design (individual randomization, parallel groups) accounted for in the conduct and analysis of the trial?

①Is it clear in the study what is the cause and what is the effect.②Were the participants included in any comparisons similar?③Were the participants included in any comparisons receiving similar treatment/care, other than the exposure or intervention of interest?④Was there a control group?？⑤Were there multiple measurements of the outcome both pre and post the intervention/exposure?⑥Was follow-up complete and if not,were differences between groups in terms of their follow up adequately described and analyzed?⑦Were the outcomes of participants included in any comparisons measured in the same way?⑧Were outcomes measured in a reliable way?⑨Was appropriate statistical analysis used？
